# Melatonin-Mediated Suppression of mtROS-JNK-FOXO1 Pathway Alleviates Hypoxia-Induced Apoptosis in Porcine Granulosa Cells

**DOI:** 10.3390/antiox12101881

**Published:** 2023-10-19

**Authors:** Xuan Zhang, Dingding Zhang, Hongmin Li, Zhaojun Liu, Yatong Yang, Jiameng Li, Lishiyuan Tang, Jingli Tao, Honglin Liu, Ming Shen

**Affiliations:** Department of Animal Genetics, Breeding and Reproduction, College of Animal Science and Technology, Nanjing Agricultural University, Nanjing 210095, China; 2021205011@stu.njau.edu.cn (X.Z.); 2020205002@stu.njau.edu.cn (D.Z.); 2021105018@stu.njau.edu.cn (H.L.); 2021205003@stu.njau.edu.cn (Z.L.); 15121315@stu.njau.edu.cn (Y.Y.); 35121110@stu.njau.edu.cn (J.L.); 15121322@stu.njau.edu.cn (L.T.); taojingli@njau.edu.cn (J.T.); liuhonglin@njau.edu.cn (H.L.)

**Keywords:** granulosa cell, mtROS, melatonin, SOD2, apoptosis

## Abstract

Numerous studies have established that the hypoxic conditions within ovarian follicles induce apoptosis in granulosa cells (GCs), a pivotal hallmark of follicular atresia. Melatonin (N-acetyl-5-methoxytryptamine, MT), a versatile antioxidant naturally present in follicular fluid, acts as a safeguard for maintaining GCs’ survival during stress exposure. In this study, we unveil an innovative protective mechanism of melatonin against hypoxia-triggered GC apoptosis by selectively inhibiting mitochondrial ROS (mtROS) generation. Specifically, under hypoxic conditions, a gradual accumulation of mitochondrial ROS occurred, consequently activating the JNK-FOXO1 pathway, and driving GCs toward apoptosis. The blocking of JNK or FOXO1 diminished hypoxia-induced GC apoptosis, but this effect was nullified in the presence of GSH, indicating that mtROS instigates apoptosis through the JNK-FOXO1 pathway. Consistent with this, hypoxic GCs treated with melatonin exhibited decreased levels of mtROS, reduced JNK-FOXO1 activation, and mitigated apoptosis. However, the protective capabilities of melatonin were attenuated upon inhibiting its receptor MTNR1B, accompanied by the decreased expression of antioxidant genes. Notably, SOD2, a key mitochondrial antioxidant gene modulated by the melatonin–MTNR1B axis, effectively inhibited the activation of mtROS-JNK-FOXO1 and subsequent apoptosis, whereas SOD2 knockdown abrogated the protective role of melatonin in hypoxic GCs. In conclusion, our study elucidates that melatonin, through MTNR1B activation, fosters SOD2 expression, effectively quelling mtROS-JNK-FOXO1-mediated apoptosis in follicular GCs under hypoxic stress.

## 1. Introduction

Ovaries, the primary reproductive organs in female mammals, contain numerous functional units known as ovarian follicles at various stages of development [1]. Abnormal follicle development can lead to long-term anovulation, menstrual disorders, infertility, and an increased risk of endometrial cancer [2]. The follicle consists of a single oocyte surrounded by granulosa cells (GCs). Under physiological conditions, follicles grow and develop in a low-oxygen environment. The follicular basal membrane acts as a barrier, preventing blood vessels from entering the follicle and limiting the transport of oxygen into the follicular interior. As a result, the oxygen partial pressure within the follicle decreases, creating a hypoxic microenvironment for GCs. Hypoxia emerges as a critical factor driving the apoptosis of GCs. The extensive apoptosis of GCs is known to play a significant role in follicular atresia, ultimately leading to the degeneration and loss of follicles from the ovaries [3,4].

Reactive oxygen species (ROS) are chemically reactive molecules containing oxygen that are generated as natural byproducts of cellular aerobic metabolism. Mitochondria, the energy-producing organelles of the cell, are particularly notable as a significant source of ROS [5]. The mitochondria-derived ROS, commonly referred to as mtROS, can significantly impact cellular functions by interacting with molecules such as DNA, proteins, and lipids [6]. When exposed to hypoxia, where oxygen is limited as an electron acceptor in the mitochondrial electron transport chain (ETC), there is a substantial buildup of mtROS. This buildup can lead to oxidative damage in cells, potentially resulting in cellular harm and apoptosis in GCs. Nevertheless, further investigation is required to clarify the correlation between the accumulation of mitochondrial ROS and the initiation of apoptosis in GCs under hypoxic conditions. In the ovaries, an excessive accumulation of ROS disrupts the redox balance, leading to oxidative stress. Substantial evidence suggests that oxidative stress plays a primary role in causing various follicular development disorders and reproductive impairments. For instance, studies have shown that knocking down the superoxide dismutase 1 (*SOD1*) gene in mice leads to mtROS accumulation and a reduction in the proportion of pre-ovulatory follicles and corpus luteum [7]. Similarly, oxidative stress resulting from glutathione (GSH) deficiency causes damage to GCs and accelerates ovarian aging [8]. Conversely, inhibiting mtROS production within follicles has been demonstrated to significantly reduce GC apoptosis and mitigate follicular atresia induced by oxidative stress [9,10]. These findings provide further substantiation for oxidative stress as a pivotal contributor to follicular atresia and GC apoptosis.

In follicular fluid, there are numerous endogenous antioxidants, with melatonin being particularly abundant. Melatonin is an indole hormone widely present in mammalian organisms, primarily secreted by the pineal gland, and also by certain local cells and tissues [11,12]. Recently, accumulated evidence has revealed the occurrence of melatonin synthesis in GCs, cumulus cells, and oocytes in the ovaries [13,14,15]. These observations suggest a potential role for melatonin synthesized locally, in conjunction with circulating melatonin, in preserving follicular health and facilitating the maturation of specific follicles. However, there is still a fundamental lack of understanding regarding the mechanisms underlying the regulation of GCs and follicular hypoxic damage by melatonin. 

In addition to its inherent antioxidative activity, melatonin can indirectly exert antioxidant effects by binding to receptors such as membrane receptors MTNR1A, MTNR1B, and nuclear receptor ROR, thereby activating the expression of antioxidant genes such as *SOD2*, *CAT*, *GPX4*, *GR*, and *GSH*. Of particular importance, *SOD2*, also known as *MnSOD*, is uniquely situated within mitochondria. Its strategic positioning enables it to effectively target and eradicate the generation of mtROS [16,17], thus offering substantial cellular protection against oxidative stress. In essence, this suggests that melatonin has the potential to directly impede the generation of mtROS at its point of origin, thereby curbing the initiation of apoptotic pathways further downstream.

Understanding the precise molecular mechanisms through which melatonin counteracts hypoxia-induced apoptosis in porcine GCs may lay the groundwork for advancing techniques and interventions aimed at promoting follicular development and ameliorating reproductive disorders. Thus, this study aimed to investigate whether mtROS induces apoptosis in porcine GCs through the JNK-FOXO1 pathway and ultimately determine if melatonin may inhibit hypoxia-induced apoptosis in these cells via the mtROS-JNK-FOXO1 pathway.

## 2. Materials and Methods

### 2.1. Reagents and Antibodies

Cell culture medium Dulbecco’s modification of Eagle’s medium/F12 (DMEM/F12), fetal bovine serum (FBS), phosphate buffer saline (PBS), and other related reagents were purchased from Gibco (Grand Island, NY, USA). Melatonin (M5250), DMSO (D8418), 4P PDOT (SML1189) and GSH (G6013) were obtained from Sigma-Aldrich (St. Louis, MO, USA), while SP600125 (S1460) and AS1842856 (S8222) were obtained from Selleck Chemicals (Houston, TX, USA).

The following antibodies were used in the study: Foxo1 (1:1000, Cell Signaling Technology, Danvers, MA, USA, 2880), p-FOXO1 (1:1000, Cell Signaling Technology, 84192), JNK (1:1000, Cell Signaling Technology, 9252), p-JNK (1:1000, Cell Signaling Technology, 4668), Caspase-3 (1:1000, Proteintech, Chicago, IL, USA, 19677-1-AP), BAX (1:1000, Proteintech, 50599-2-Ig), BCL2 (1:1000, Proteintech, 68103-1-Ig), TUBA1A (1:2000, Cell Signaling Technology, 2125), Anti-Rabbit IgG H&L (HRP), 1:2000, Abcam, Cambridge, UK, ab6721; Goat Anti-Mouse IgG H&L (HRP), 1:2000, Abcam, ab6789.

### 2.2. Sample Collection and Cell Culture

In this study, ovaries were obtained from mature Duroc–Yorkshire–Landrace sows at Nanjing Zhushun Biotechnology Co., Ltd. (Nanjing, China) and were promptly transported to the laboratory. All experiments and treatments received approval from the Animal Research Institute Committee at Nanjing Agricultural University, China, under permit number SYXK2022-0031. To isolate GCs, we employed a previously established method [18]. Specifically, we selected ovaries at the follicular stage and collected granulosa cells from medium-sized follicles (3–5 mm) for culture. Subsequently, they were seeded onto cell culture plates and cultured in DMEM/F12 medium containing 15% FBS at 37 °C with 5% CO_2_. After 36 h, the GCs adhered to the plate. Non-adherent impurities were washed off with PBS, and then the cells were cultured in a medium containing 10% FBS. When the cell confluence reached above 70%, further experiments were conducted.

### 2.3. Experimental Design and Cell Treatment

The experimental design and grouping are outlined as follows:

(1) Impact of hypoxia treatment on GCs’ mitochondrial ROS production: Porcine GCs were cultured at 37 °C in an environment containing 1% O_2_ (Hypoxia) or 21% O_2_ (Normoxia) using the previously established method [1,19]. mtROS levels were measured at 6–36 h after treatment.

(2) Elucidation of whether mtROS induces GC apoptosis via activation of the JNK-FOXO1 pathway: We assessed whether the JNK-FOXO1 pathway was activated by adding the ROS scavenger (GSH, 2 mM). Additionally, cells were pre-treated with a JNK inhibitor (SP600125, 10 μM) or FOXO1 inhibitor (AS1842856, 10 μM) for 2 h, or FOXO1 was knocked down by siRNA for 24 h, followed by GSH treatment to determine JNK-FOXO1 activation. The specific experimental groups included: Normoxia, Hypoxia, Hypoxia + GSH, Hypoxia + SP600125, Hypoxia + GSH + SP600125, Hypoxia + si*FOXO1*, Hypoxia + GSH + si*FOXO1*, Hypoxia + AS1842856, and Hypoxia + GSH + AS1842856.

(3) Impact of melatonin treatment on mitochondrial ROS production in hypoxic GCs: GCs were cultured under hypoxic conditions and treated with varying concentrations (10^−9^ to 10^−3^ M) of exogenous melatonin for 36 h. Following that, we evaluated mtROS levels and determined that a concentration of 10^−5^ M melatonin would be used for subsequent experiments. To investigate changes in apoptosis levels, a combination of GSH and melatonin was added for 24 h.

(4) Exploration of upstream pathways of melatonin in mitigating mtROS: To investigate whether melatonin affects mtROS generation via the MTNR1B receptor, we employed 4P PDOT (100 nM), a potent and selective MTNR1B antagonist. Cells were divided into four groups: Normoxia, Hypoxia, Hypoxia + MT, and Hypoxia + MT + 4P. The impact on mtROS, antioxidant gene expression, and the JNK-FOXO1-apoptosis pathway was evaluated. Furthermore, SOD2, a specific mitochondrial ROS scavenger, was employed to investigate whether melatonin clears mtROS via the MTNR1B-SOD2 pathway. Cells were divided into several groups: Hypoxia, Hypoxia + MT, Hypoxia + OE-*SOD2*, and Hypoxia + MT + si-*SOD2*, and mtROS levels and regulation of the JNK-FOXO1 pathway proteins were assessed.

### 2.4. Mitochondrial ROS Detection

After the GCs were treated as described in the study, mitochondrial ROS (mtROS) levels in each group were assessed using the MitoSOX Red probe from Yeasen (Shanghai, China), following the manufacturer’s instructions. The cells were incubated at 37 °C in the dark for 10 min, followed by three washes with PBS. Next, 50 nM MitoTracker™ Green FM (ThermoFisher, Waltham, MA, USA, M7514) was added, and the cells were further incubated at 37 °C in the dark for 30 min. After three additional washes with PBS, ROS levels in the GCs were visualized and captured using a laser-scanning confocal microscope (LSM700ME-TA; Zeiss, Oberkochen, Germany). Subsequently, the obtained results were quantified and analyzed for each group using ImageJ 1.42q software.

### 2.5. Western Blotting

After treating the GCs as described in this study, we harvested total protein using RIPA lysis buffer (Beyotime, Shanghai, China). Subsequently, we loaded 20 μg of denatured protein, equally divided per lane, onto a 4–20% SDS-PAGE gel and conducted electrophoresis. The proteins were then transferred onto a polyvinylidene difluoride (PVDF) membrane (Millipore, Bedford, MA, USA) through electrotransfer. Next, the PVDF membrane was blocked with 5% bovine serum albumin (BSA, Sigma-Aldrich, A3059) at 37 °C for 60 min, followed by an overnight incubation at 4 °C with primary antibodies. After washing the blots with Tween-20 (TBST) buffer, they were incubated with secondary antibodies. Following three washes with TBST buffer, the membranes underwent chemiluminescence detection. Finally, all the blots were analyzed using Image J software (version 1.51k), and the values for the target proteins were normalized to TUBA1A, which served as the control.

### 2.6. Immunofluorescence

After the GCs were treated as described in this study, the cells were fixed with 4% paraformaldehyde at room temperature for 1 h. Following three washes with PBS, the cells were permeabilized with 0.5% Triton X-100 (Sigma-Aldrich, T8787) at 4 °C for 10 min. After another three washes with PBS, the cells were blocked with 5% BSA at room temperature for 1 h. Subsequently, the cells were incubated with a 1:100 dilution of FOXO1 antibody at 4 °C overnight. After three washes with PBS, the cells were incubated with rhodamine-conjugated goat anti-rabbit/mouse IgG (H + L) antibody (dilution 1:100; ZSGB-BIO, Beijing, China) at room temperature for 1 h, followed by three additional washes with PBS. The cells were then stained with DAPI (Keygen, Nanjing, China) at 37 °C for 10 min in the dark and washed with PBS three times. Finally, the slides were mounted with a fluorescence quenching agent (Biosharp, Hefei, China), and images were captured using a confocal microscope (LSM700ME-TA; Zeiss, Oberkochen, Germany).

### 2.7. TUNEL Assay

After treatment, the GCs seeded on 24-well cell culture plates were subjected to apoptosis detection using the TUNEL assay kit (Yeasen, Shanghai, China). The specific steps were as follows: the treated GCs were washed twice with PBS, fixed in 4% paraformaldehyde at 4 °C for 25 min, and then washed three times with PBS. Subsequently, the cells were incubated with 0.2% Triton X-100 at room temperature for 5 min and washed three times with PBS. Next, the cells were incubated at room temperature for 30 min in 1× equilibration buffer, followed by the addition of 50 μL TdT incubation buffer per well. The cells were then incubated at 37 °C in a humidified chamber in the dark for 1 h. After washing three times with PBS, the GCs were incubated at room temperature in a 2 μg/mL DAPI solution for 5 min in the dark. Finally, the cells were washed three times with PBS and imaged using confocal microscopy (LSM700ME-TA; Zeiss, Oberkochen, Germany).

### 2.8. Quantitative Real-Time PCR (qRT-PCR)

After processing all GCs from each group described above, total RNA was isolated using Trizol reagent (Invitrogen, Waltham, MA, USA). Subsequently, cDNA was synthesized from 1 μg of RNA per sample using the PrimeScript RT Reagent Kit (Yeasen, China) following the manufacturer’s protocols.

The qRT-PCR was performed using Hieff qPCR SYBR Green Master Mix (Yeasen, China) to evaluate gene expression levels. The steps for real-time quantitative PCR (qRT-PCR) were as follows: employing a two-step amplification method, an initial denaturation at 95 °C for 30 s, followed by denaturation at 95 °C for 10 s, annealing at 60 °C for 30 s, and concluding with a melting curve analysis. *TUBA1A* was used as the endogenous control for samples. The primer sequences used in the qRT-PCR are listed in Table 1. The relative gene expression levels were analyzed using the 2^−ΔΔCt^ method.

### 2.9. RNA Interference and Gene Overexpression

The knockdown of *FOXO1* and *SOD2* was performed using synthetic siRNA synthesized by Qingke (Nanjing, China), with a scrambled siRNA serving as a control. GCs were transfected with gene-specific siRNA or the control siRNA (50 nM) for 24 h using Lipofectamine 3000 (Thermo Fisher Scientific, Waltham, MA, USA) according to the manufacturer’s instructions. Additionally, the OE-*SOD2* plasmid, also synthesized by Qingke, was used. Similarly, GCs were transfected with OE-*SOD2* for 24 h using Lipofectamine 3000.

The sequence information of siRNA is as follows:

Scrambled siRNA: Sense (5′-UUCUCCGAACGUGUCACGUTT-3′)
        Antisense (5′-ACGUGACACGUUCGGAGAATT-3′)
si*FOXO1*: Sense (5′-GCAUGUUCAUUGAGCGCUUTT-3′)
    Antisense (5′-AAGCGCUCAAUGAACAUGCTT-3′)
si*SOD2*: Sense (5′-GGCCACAUCAAUCAUAGCATT-3′)
   Antisense (5′-UGCUAUGAUUGAUGUGGCC-3′)

### 2.10. Statistics Analysis

All data were presented as means ± S.E.M. Analysis was performed using SPSS version 21.0 software (SPSS, Inc., Chicago, IL, USA) and GraphPad Prism version 8.0 statistical software (GraphPad, Inc., La Jolla, CA, USA). Differences between two groups were assessed using the Student *t*-test, while comparisons involving more than two groups were analyzed using one-way analysis of variance (ANOVA). All experiments were repeated at least three times. Horizontal bars represent treatments with significant differences at *p* < 0.05.

## 3. Results

### 3.1. Hypoxia Promotes GC Apoptosis by Increasing the Accumulation of mtROS

**Figure 1:**
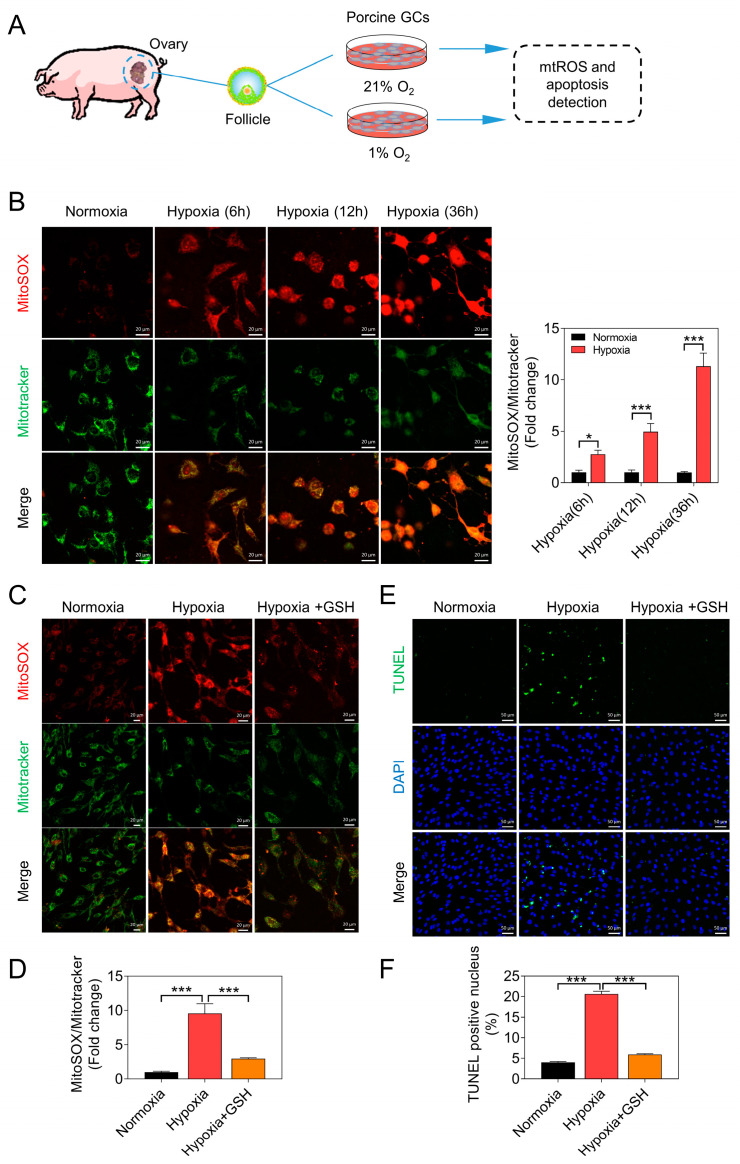
Hypoxia promotes apoptosis of GCs by increasing the accumulation of mtROS. (**A**) Summary of the experimental design. (**B**) Representative images display MitoSOX Red fluorescence and MitoTracker Green fluorescence. Scale bar = 20 μm. The ratio of MitoSOX to MitoTracker is presented on the right side. (**C**) Representative images of MitoSOX Red fluorescence and MitoTracker Green fluorescence. Scale bar = 20 μm. (**D**) Quantification of the fluorescence ratio of MitoSOX to MitoTracker. (**E**) After a 24 h treatment of GCs, TUNEL staining was utilized to indicate cells undergoing apoptosis. Scale bar = 50 μm. (**F**) Quantification of the ratio of TUNEL-positive staining cells. Data are presented as means ± S.E.M. *p* < 0.05 *, *p* < 0.001 ***.

We assessed mtROS levels in GCs cultured under hypoxic conditions for varying durations. The results demonstrated a time-dependent increase in mtROS levels with prolonged hypoxia incubation (Figure 1A,B). To evaluate the impact of mtROS on GC apoptosis, we introduced the ROS scavenger, GSH. Experimental results revealed that under hypoxic conditions, the addition of GSH effectively cleared the intracellular accumulation of mtROS (Figure 1C,D). Furthermore, GSH exhibited a concurrent inhibitory effect on the hypoxia-induced apoptosis of GCs (Figure 1E,F).

### 3.2. Hypoxia Induces GC Apoptosis by Activating the mtROS-JNK-FOXO1 Pathway

**Figure 2:**
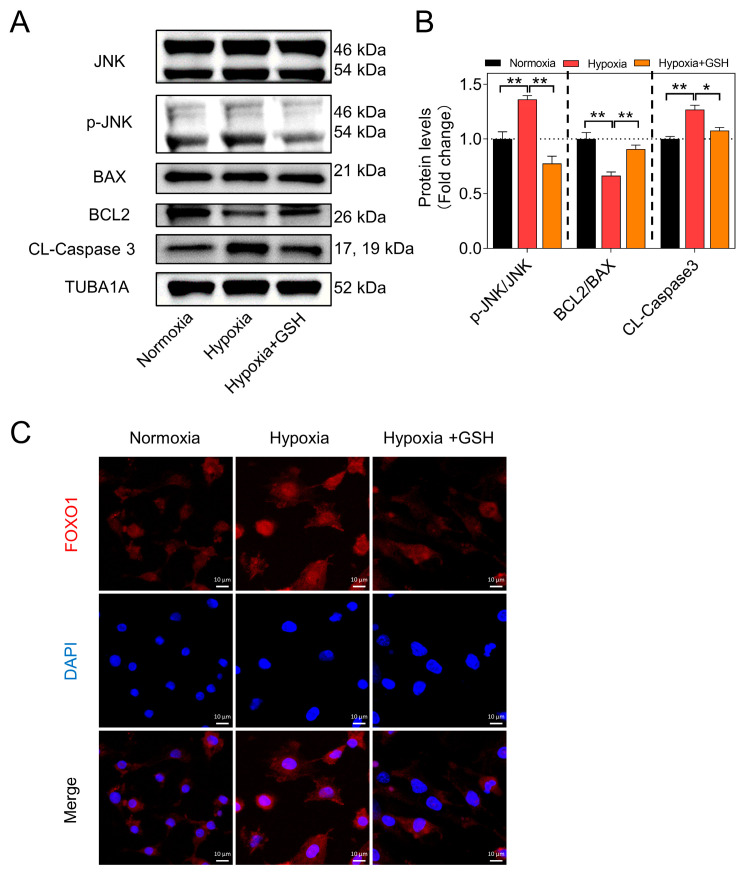
Hypoxia induces GC apoptosis by activating the mtROS-JNK-FOXO1 pathway. (**A**) The levels of JNK, p-JNK, BAX, BCL2, and Cleaved-Caspase3 of GCs in each group were detected by Western blotting. (**B**) The protein levels were normalized with TUBA1A. (**C**) FOXO1 (red) nuclear localization was assessed using immunofluorescence, with the nucleus counterstained using DAPI (blue). Scale bar = 10 μm. Data are presented as means ± S.E.M. *p* < 0.05 *, *p* < 0.01 **.

Previous studies have demonstrated that the core mechanism of GC apoptosis induced by oxidative stress involves the activation of the JNK-FOXO1 pathway [19]. To investigate this, we conducted Western blotting to detect the levels of JNK and apoptosis-related proteins. The results indicated that GSH treatment led to a reduction in the levels of apoptosis-related proteins, such as Cleaved-Caspase3, and a significant increase in the BCL2/BAX ratio (Figure 2A,B). Furthermore, the translocation of FOXO1 into the nucleus was notably inhibited (Figure 2C).

**Figure 3:**
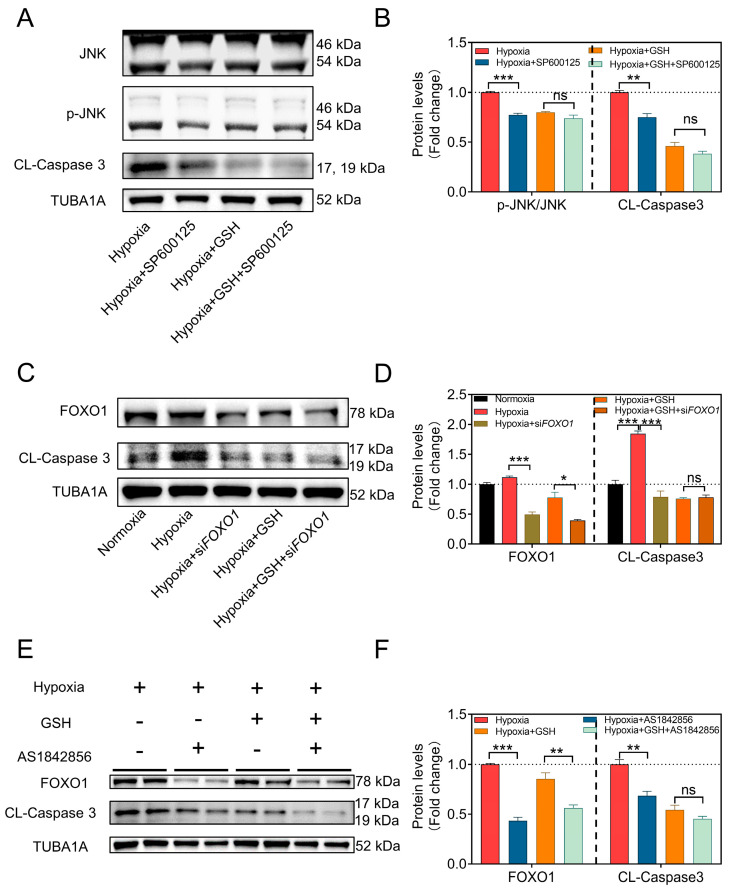
Blocking the JNK-FOXO1 pathway alleviates mtROS-induced GC apoptosis. (**A**) After pre-treating GCs with the JNK inhibitor SP600125 for 2 h, they were further treated with GSH or PBS for 12 h. Subsequently, the protein levels of JNK, p-JNK, and Cleaved-Caspase3 were assessed using Western blot, (**B**) followed by grayscale analysis. (**C**) After knocking down GCs using siRNA for 12 h, they were further treated with GSH or PBS for 12 h. Subsequently, the protein levels of FOXO1 and Cleaved-Caspase3 were assessed using Western blot, (**D**) followed by grayscale analysis. (**E**) After pre-treating GCs with the FOXO1 inhibitor AS1842856 for 2 h, they were further treated with GSH or PBS for 12 h. Subsequently, the protein levels of FOXO1 and Cleaved-Caspase3 were assessed using Western blot, (**F**) followed by grayscale analysis. Data are shown as mean ± SEM. ns, not significance, *p* < 0.05 *, *p* < 0.01 **, *p* < 0.001 ***.

For further investigation, we initially introduced the JNK inhibitor SP600125. The results showed that when JNK phosphorylation was inhibited, the levels of the apoptosis execution protein Cleaved-Caspase3 significantly decreased. However, the co-addition of SP600125 and GSH did not show a significant difference in apoptotic levels compared to the addition of GSH alone (Figure 3A,B). Next, we employed two strategies to investigate whether mtROS influences GC apoptosis through FOXO1. First, we used siRNA to knock down FOXO1 expression and found that inhibiting FOXO1 expression under hypoxic conditions suppressed cell apoptosis. Moreover, the addition of AS1842856 (a direct inhibitor of activated FOXO1 that inhibits its transactivation) yielded similar results. These findings demonstrated that inhibiting FOXO1 expression or activity under hypoxic conditions resulted in the suppression of cell apoptosis, and GSH-mediated mtROS clearance produced similar results (Figure 3C–F). Particularly, the suppression of FOXO1 provided no additional protective effects when GCs were pretreated with AS1842856 and/or GSH. These data indicate that the accumulation of mtROS in GCs is promoted under hypoxia, which further activates the JNK-FOXO1 pathway, leading to FOXO1 translocation to the nucleus, and eventually inducing GC apoptosis.

### 3.3. Melatonin Alleviates Hypoxia-Induced Apoptosis of GCs by Reducing mtROS Levels

**Figure 4:**
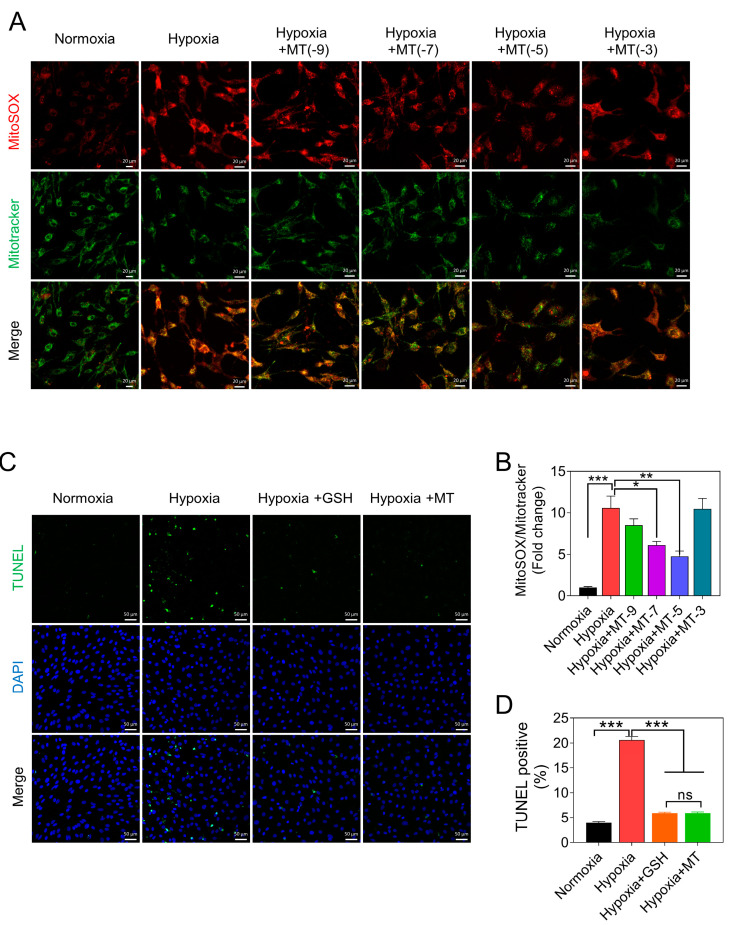
Blocking the JNK-FOXO1 pathway alleviates mtROS-induced GC apoptosis. (**A**) Representative images of MitoSOX Red fluorescence and MitoTracker Green fluorescence. Scale bar = 20 μm. (**B**) The ratio of MitoSOX/MitoTracker. (**C**) After a 24 h treatment of GCs, TUNEL staining was utilized to indicate cells undergoing apoptosis. Scale bar = 50 μm. (**D**) Quantification of the ratio of TUNEL-positive staining cells. Data are shown as means ± S.E.M. ns, not significance, *p* < 0.05 *, *p* < 0.01 **, *p* < 0.001 ***.

To elucidate the effect of melatonin on mtROS levels in GCs under hypoxic conditions, we added melatonin at concentrations ranging from 10^−9^ to 10^−3^ mol/L. We observed a significant reduction in mtROS levels at concentrations ranging from 10^−9^ to 10^−5^ mol/L (Figure 4A,B). Notably, melatonin exhibited a significant increase in mtROS levels at a concentration of 10^−3^ mol/L (Figure 4A,B). Therefore, in subsequent experiments, we maintained the melatonin treatment concentration at 10^−5^ mol/L. Subsequently, TUNEL staining results demonstrated that melatonin significantly reduced hypoxia-induced GC apoptosis (Figure 4C,D). Noteworthily, melatonin exerted a comparable effect to GSH on the apoptosis of GCs (Figure 4C,D).

### 3.4. Melatonin Inhibits Hypoxia-Induced mtROS Generation and GC Apoptosis through the MTNR1B Receptor

**Figure 5:**
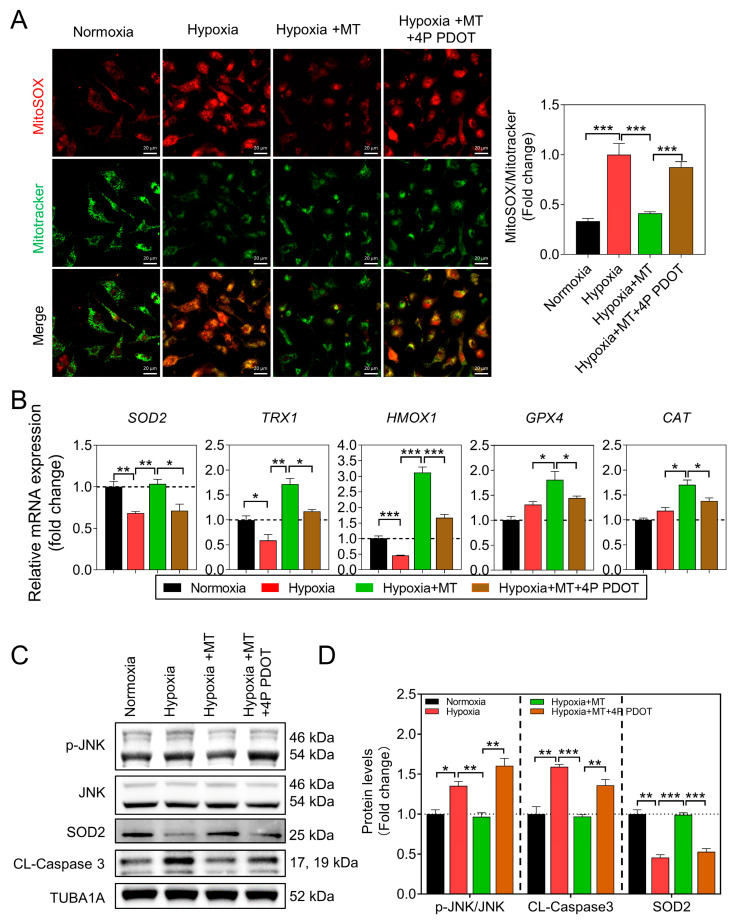
Melatonin alleviates the accumulation of mtROS and inhibits JNK activation through the MTNR1B receptor in GCs. (**A**) Representative images of MitoSOX Red fluorescence and MitoTracker Green fluorescence. Scale bar = 20 μm. The ratio of MitoSOX to MitoTracker fluorescence is presented on the right side. (**B**) Quantification of antioxidant enzyme gene expression in GCs following treatment using qRT-PCR. (**C**) After pre-treating GCs with the MTNR1B inhibitor 4P PDOT for 2 h, they were further treated with MT or DMSO for 12 h. Subsequently, the protein levels of JNK, p-JNK, SOD2, and Cleaved-Caspase3 were assessed using Western blot. (**D**) The protein levels were normalized with TUBA1A. Data are shown as means ± S.E.M. *p* < 0.05 *, *p* < 0.01 **, *p* < 0.001 ***.

Previous studies have demonstrated that the anti-apoptotic effect of melatonin in GCs is mediated through the MTNR1B receptor [20,21,22]. In this study, we used 4P PDOT, a potent and selective MTNR1B antagonist, to inhibit the MTNR1B receptor, resulting in the abolition of the melatonin-mediated suppression of mtROS formation (Figure 5A). Furthermore, we examined the transcription levels of antioxidant genes and found that melatonin counteracts the inhibitory effect of hypoxia on the expression of antioxidant-related genes, such as *SOD2*, *TRX1*, *HMOX1*, *GPX4*, and *CAT* (Figure 5B). Notably, SOD2, an antioxidant enzyme located in mitochondria, plays a crucial role in scavenging mtROS. Under hypoxic conditions, there was a significant reduction in *SOD2* expression, but melatonin significantly enhanced its transcriptional activity (Figure 5B), implying that *SOD2* might play a pivotal role in mitigating mtROS levels by melatonin in response to hypoxia in GCs. In addition, 4P PDOT inhibited the phosphorylation of JNK and reversed the promotion of *SOD2* expression by melatonin, as well as the reduction in Cleaved-Caspase3 protein levels (Figure 5B–D).

**Figure 6:**
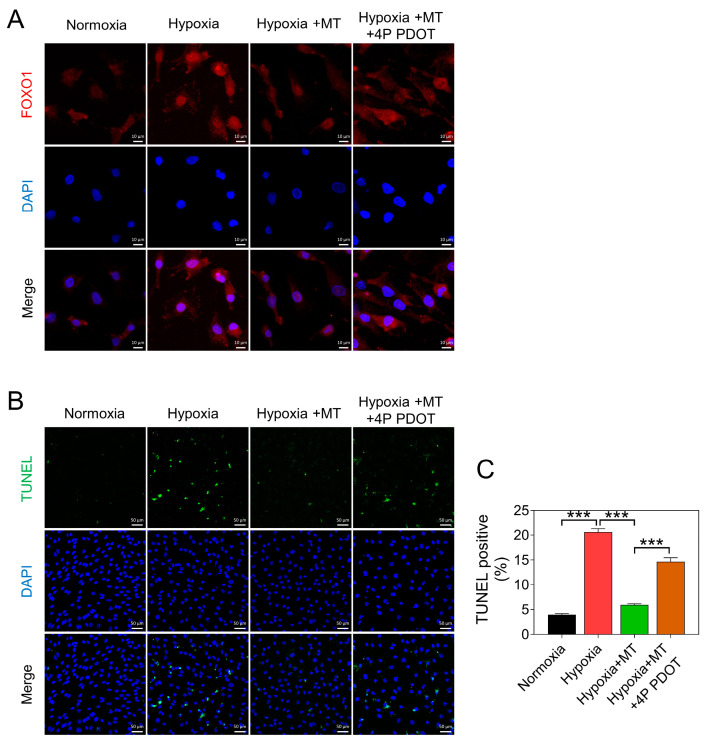
Melatonin inhibits hypoxia-induced nuclear translocation of FOXO1 and apoptosis through MTNR1B. (**A**) FOXO1 (red) nuclear localization was assessed using immunofluorescence, with the nucleus counterstained using DAPI (blue). Scale bar = 10 μm. (**B**) After a 24 h treatment of GCs, TUNEL staining was utilized to indicate cells undergoing apoptosis. Scale bar = 50 μm. (**C**) Quantification of the ratio of TUNEL-positive staining cells. Data are shown as means ± S.E.M. *p* < 0.001 ***.

Moreover, we observed that the melatonin-induced upregulation of SOD2 protein levels in hypoxic GCs was significantly reduced upon MTNR1B inhibition (Figure 5C,D). Consistent with these results, we found that the inhibitory effect of melatonin on hypoxia-induced FOXO1 nuclear translocation was also relieved after blocking MTNR1B (Figure 6A). Similarly, the TUNEL staining assay showed that the inhibitory effect of melatonin on hypoxia-induced GC apoptosis was attenuated after MTNR1B inhibition (Figure 6B).

### 3.5. SOD2 Contributes to the Inhibition of Hypoxia-Induced mtROS Accumulation and GC Apoptosis by Melatonin

**Figure 7:**
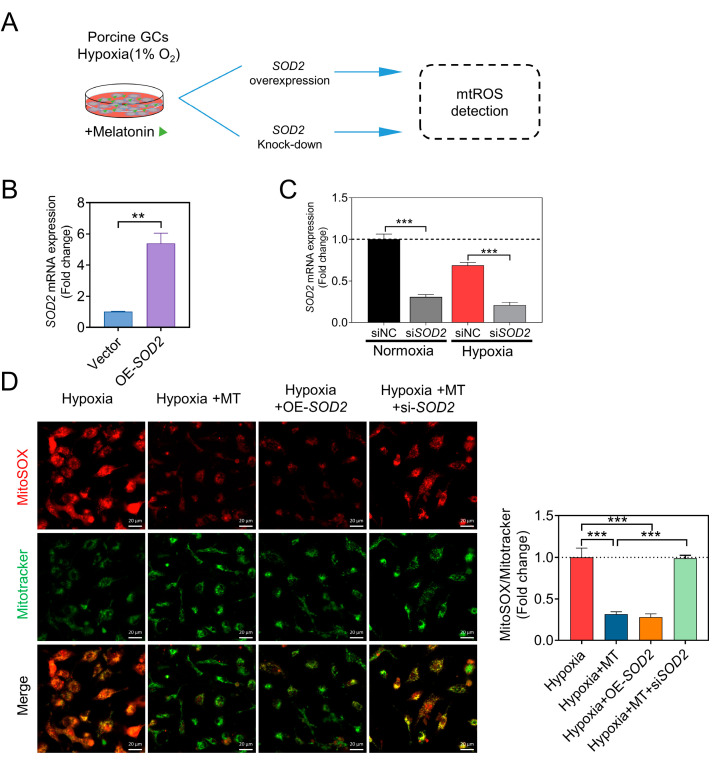
Melatonin reduces the accumulation of mtROS through SOD2. (**A**) Overview of the experimental design. (**B**) Quantitative real-time PCR was employed to detect the transcription levels of the SOD2 gene after overexpression. (**C**) Quantitative real-time PCR was utilized to assess the transcription levels of the SOD2 gene following knockdown. (**D**) Representative images display MitoSOX Red fluorescence and MitoTracker Green fluorescence. Scale bar = 20 μm. The ratio of MitoSOX to MitoTracker fluorescence is presented on the right side. Data are shown as means ± S.E.M. *p* < 0.01 **, *p* < 0.001 ***.

After overexpressing or knocking down *SOD2* in GCs, we observed a substantial elevation in mRNA levels in the OE-*SOD2* group (Figure 7A,B). Conversely, SOD2 knockdown led to a notable reduction in *SOD2* expression levels, both in normoxic and hypoxic conditions (Figure 7C). However, when *SOD2* was knocked down after melatonin treatment, the clearance of mtROS by melatonin was reversed (Figure 7D).

**Figure 8:**
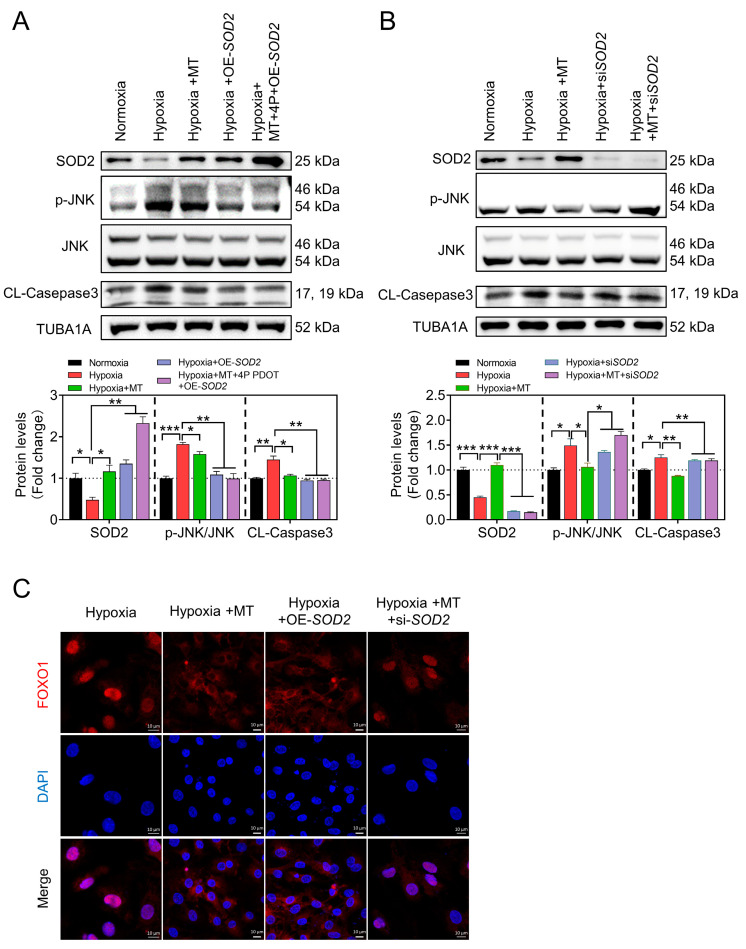
Melatonin alleviates hypoxia-induced mtROS-JNK-FOXO1 pathway activity and apoptosis through SOD2 in GCs. (**A**,**B**) Protein levels of JNK, p-JNK, SOD2, and Cleaved-Caspase3 were assessed using Western blotting, with protein levels normalized using TUBA1A. (**C**) FOXO1 (red) nuclear localization was assessed using immunofluorescence, with the nucleus counterstained using DAPI (blue). Scale bar = 10 μm. Data are shown as means ± S.E.M. *p* < 0.05 *, *p* < 0.01 **, *p* < 0.001 ***.

Further experiments revealed that SOD2 overexpression restored SOD2 protein levels, resulting in a significant decrease in JNK activity and Cleaved-Caspase3 levels under hypoxic conditions (Figure 8A). Additionally, when we blocked the MTNR1B receptor using 4P PDOT in melatonin-treated GCs, the overexpression of SOD2 could still effectively inhibit the apoptosis of GCs upon hypoxia exposure. In contrast, knocking down SOD2 undermined the inhibitory effects of melatonin on JNK activation and Caspase3 cleavage (Figure 8B). Moreover, the subcellular localization analysis of FOXO1 revealed that the OE-SOD2 group prominently hindered FOXO1 nuclear translocation under hypoxic conditions, while SOD2 knockdown counteracted the inhibitory effect of melatonin on FOXO1 nuclear translocation (Figure 8C).

## 4. Discussion

**Figure 9:**
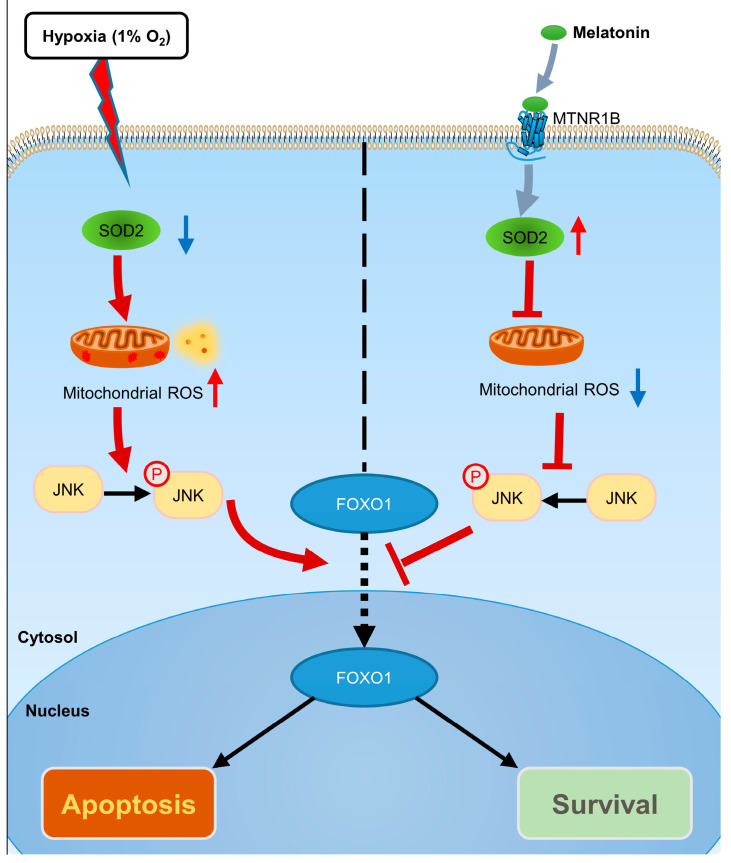
A schematic model depicts melatonin-mediated GC protection against hypoxic damage via activating the MTNR1B-SOD2-mtROS-JNK-FOXO1 axis. Under hypoxic conditions, decreased SOD2 levels and the accumulation of mitochondrial ROS contribute to the activation of the JNK-FOXO1 pathway, ultimately inducing GC apoptosis. In the presence of melatonin, it binds to the MTNR1B receptor and promotes SOD2 expression, facilitating the clearance of accumulated mtROS, thereby abrogating the activation of the JNK-FOXO1 pathway, and consequently inhibiting hypoxia-induced GCs apoptosis. Upregulation is represented by the thin red arrow, while downregulation is denoted by the thin blue arrow.

The current study offers a comprehensive insight into the protective mechanism of melatonin against hypoxic stress in porcine GCs. We meticulously identified mtROS as a primary source of oxidative damage in GCs exposed to hypoxia. Furthermore, we elucidated the fundamental mechanism by which mtROS triggers apoptosis in GCs through the activation of the JNK-FOXO1 pathway. Notably, our findings unveil that melatonin plays a pivotal role in orchestrating the clearance of mtROS. This regulatory effect is achieved through the modulation of the expression of the mitochondrial-specific antioxidant enzyme, SOD2. Consequently, the regulatory mechanism effectively suppresses the mtROS-JNK-FOXO1 pathway, ultimately leading to a significant attenuation of apoptosis in GCs subjected to hypoxic stress (refer to Figure 9).

Maintaining an optimal intracellular redox balance is vital for cell survival, as excessive accumulation of ROS can disrupt this equilibrium, resulting in oxidative stress, a prominent contributor to cell apoptosis. Research across various species, including cattle [23], sheep [24], pigs [1,25,26], mice [27,28], and human follicles [29], has consistently shown that oxidative stress detrimentally affects GCs within mammalian follicles. Our previous investigations have also unveiled a direct correlation between elevated ROS accumulation and increased rates of apoptosis in GCs [1,25]. Endogenous sources of ROS generation encompass NOX, hypoxia, metabolic disturbances, and endoplasmic reticulum stress [30,31]. Regarding hypoxia, previous studies have predominantly focused on its impact on overall intracellular ROS levels in GCs, thereby facilitating cellular apoptosis [32,33,34]. However, the precise sources of ROS and the underlying mechanisms triggering apoptosis in this context have remained elusive. In this study, we present pioneering evidence that the accumulation of mtROS serves as the fundamental trigger for hypoxia-induced apoptosis in GCs.

Existing reports highlight the activation of the JNK-FOXO1 pathway as a pivotal mechanism in hypoxia-induced apoptosis of GCs [19]. However, the upstream signaling molecules contributing to this pathway remain largely unexplored and necessitate further investigation. Intriguingly, activation of the JNK-FOXO1 pathway is also central to the mechanism of apoptosis induced by oxidative stress in GCs. In support of this, Yang et al. demonstrated that oxidative stress prompts JNK activation, leading to GC apoptosis, and the introduction of JNK inhibitors effectively reduces cellular apoptosis [35]. Similarly, the research of Huang et al. established a compelling link between JNK phosphorylation, nuclear translocation of FOXO1, and cellular apoptosis [36]. In this study, we have successfully elucidated that hypoxia-induced apoptosis in GCs stems from the generation of mtROS, which subsequently triggers the activation of the JNK-FOXO1 pathway. Consequently, our findings establish a coherent signal transduction pathway that interlinks hypoxia, oxidative stress, and GC apoptosis.

As reported, melatonin demonstrates a significant alleviating effect on cellular damage induced by hypoxia. For instance, Luo et al. revealed that melatonin exerts cardioprotective effects by suppressing cell apoptosis through the activation of the PI3K-AKT signaling pathway in hypoxic cardiomyocytes [37]. Moreover, in human umbilical vein endothelial cells (HUVECs), exogenous melatonin has been observed to effectively mitigate excessive ROS production induced by hypoxia/reoxygenation [38]. On the other hand, melatonin plays a crucial role in regulating both follicular development and apoptosis of GCs. In mammals, the ovarian follicular environment provides an ideal fluid milieu for accommodating melatonin secreted by the bloodstream and follicles, creating a natural reservoir for melatonin [14,39]. The research conducted by He et al. demonstrated that reduced melatonin levels in antral follicles correlate with an elevated rate of apoptosis of GCs. Conversely, the addition of exogenous melatonin effectively reduces the levels of apoptosis [40]. Moreover, in the aging process of female mammals, the reduction of melatonin in follicular fluid is associated with oxidative stress, decreased oocyte quality, and impaired follicular development [41,42,43]. In this study, we unveiled that the anti-apoptotic efficacy of melatonin on hypoxic GCs hinges on its proficient clearance of mtROS. Importantly, our data underscore that melatonin adeptly eliminates mtROS in a concentration-dependent manner, which is consistent with the findings of a previous study that examined the effects of various melatonin concentrations on cell viability and apoptosis in hypoxic GCs. Notably, the most significant mtROS clearance was observed at a concentration of 10^−5^ mol/L, while a concentration of 10^−3^ mol/L adversely impacted cell viability (Figure 4). In accordance with this, melatonin at 10^−5^ mol/L significantly restrained hypoxia-induced apoptosis, while an increased concentration of 10^−3^ mol/L reversed the suppressive effect on apoptosis [25].

The precise signaling pathways by which melatonin alleviates hypoxic damage in GCs remain elusive. In our earlier investigation [16], we revealed that melatonin, acting through its receptor MTNR1B, significantly reduces intracellular total ROS accumulation in GCs. There is also suggestive evidence from another study that melatonin could hinder the activation of JNK in GCs when subjected to oxidative stress [28,44]. However, the protective mechanism of melatonin in the context of hypoxic GCs remains largely unexplored. In the present study, we have taken a significant step forward in advancing our comprehension of the mechanism involved. Specifically, we have elucidated that melatonin, through its interaction with the MTNR1B receptor, triggers an upregulation in the expression of SOD2, which selectively hinders the formation of mtROS at its point of origin. Consequently, this function acts to inhibit the activation of the JNK-FOXO1 signaling pathway, thus safeguarding GCs against apoptosis induced by hypoxia. Overall, these findings define a novel mechanism by which melatonin ameliorates the detrimental effects of hypoxia on GCs, specifically involving the targeted regulation of the MTNR1B-SOD2-mtROS-JNK-FOXO1 signaling pathway.

Given the antioxidative properties of melatonin, it plays a pivotal role in alleviating a spectrum of developmental disturbances in reproductive organs. For instance, in the context of Polycystic Ovary Syndrome (PCOS), a common endocrine disorder associated with infertility and reproductive dysregulation, melatonin exhibits promising therapeutic effects. In a murine model of PCOS, melatonin administration significantly enhances oocyte quality and increases fertilization rates [45]. Furthermore, encouraging results have emerged from trials investigating melatonin as an oral treatment for PCOS patients. Continuous melatonin supplementation in PCOS patients ameliorates menstrual irregularities and mitigates the decline in steroid hormone levels, suggesting that melatonin helps restrain the pathological progression of PCOS [46]. Moreover, melatonin demonstrates significant alleviating effects on premature ovarian insufficiency (POI). Studies indicate that melatonin can mitigate mitochondrial damage and partially restore ovarian reserve function by downregulating the ERK pathway [47]. Additionally, melatonin exhibits some therapeutic potential in male reproductive disorders. In another study, supplementation of melatonin in rats was found to attenuate oxidative lipid damage, thereby inhibiting germ cell apoptosis induced by varicocele-related oxidative stress [48]. These findings elucidate that melatonin, by inhibiting oxidative stress, facilitates the amelioration of reproductive disorders. In our research, we have further uncovered the core mechanism underlying melatonin’s antioxidative capacity. It operates by binding to its receptors and subsequently activating the expression of SOD2, thereby eliminating ROS originating from mitochondria and, consequently, mitigating reproductive disturbances induced by oxidative stress.

In conclusion, our study has elucidated the pivotal role of melatonin in suppressing hypoxic stress and enhancing the survival of GCs. Significantly, our investigation has unveiled the pivotal involvement of mitochondrial-derived ROS in GC apoptosis and follicular atresia, establishing that ROS originating from the mitochondria initiates apoptosis by activating the JNK-FOXO1 pathway. Notably, melatonin emerges as an important regulator in this intricate process, enhancing the transcription and translation of SOD2 through MTNR1B receptor binding. SOD2, as an antioxidant enzyme specific to mitochondria, effectively suppresses the mtROS-JNK-FOXO1 pathway, ultimately reducing apoptosis in GCs. In summary, our research contributes to a deeper understanding of the regulatory mechanisms of melatonin in follicular development, which holds significant implications for follicular health and, by extension, broader reproductive well-being. Importantly, our study lays the foundation for the potential improvement of reproductive disorders and related diseases. It is our hope that these findings will ultimately contribute to improved therapies and outcomes for individuals affected by reproductive disorders.

## Figures and Tables

**Table 1:** Primers for qRT-PCR.

Gene Name	Primer Sequence (5′–3′)	GenBank Accession NO.
*HMOX-1*	F: CCGAGAAGGCTTTAAGCTGGT R: GGAAGTAGAGGGGCGTGTAG	NM_001004027.1
*TRX1*	F: CTTTACCTTATTGCCCGGGT	NM_214313.2
	R: GTTCACCGATTTTGTTGGCC	
*SOD2*	F: AGGCGCTGAAAAAGGGTGAT R: AAGTCGCGTTTGATGGCTTC	NM_214127.2
*CAT*	F: CACACATACCCATTCGTCACT R: CAGCCCTAACCTTCACTTACC	NM_214301.2
*GPX4*	F: ATTCTCAGCCAAGGACATCG R: CCTCATTGAGAGGCCACATT	NM_214407.1
*TUBA1A*	F: AAGAGTCGCGCTGTAAGAAG R: AATGACTGTGGGTTCCAGGTC	NM_001315710.1

## Data Availability

Data are contained within the article.
